# Supplementary material for: A large C-terminal Rad52 segment acts as a chaperone to Form and Stabilize Rad51 Filaments
Source: Nat Commun. 2025 Jul 1;16:5589. doi: 10.1038/s41467-025-60664-x (PMC12218292; doi:10.1038/s41467-025-60664-x)
Supplement: Supplementary file 1 — Supplementary Information [file 41467_2025_60664_MOESM1_ESM.pdf]

Rad52 Acts as an Assembly Chaperone to Form and Stabilize Rad51 Filaments Through a Large C-Terminus 85-Residue Segment

Emilie Ma[1,2], Fadma Lakhal[3], Eleni Litsardaki[4,5], Myriam Ruault[3], Maxime Audin[4,5], Natacha Levrier[1,2,4,5], Emilie Navarro[1,2], Mickaël Garnier[3], Laurent Maloisel[1,2], Jordane Depagne[6,7], Clémentine Brocas[6,7], Aurelien Thureau[8], Didier Busso[6,7], Xavier Veaute[6,7], Raphaël Guerois[4,5], Angela Taddei[3]*, Françoise Ochsenbein[4,5]* and Eric Coïc[1,2]*

*These authors jointly supervised this work

[1]Université Paris Cité, Inserm, CEA, Stabilité Génétique Cellules Souches et Radiations, LRGM/iRCM/IBFJ, F-92260 Fontenay-aux-Roses, France.
[2]Université Paris-Saclay, Inserm, CEA, Stabilité Génétique Cellules Souches et Radiations, LRGM/iRCM/IBFJ, F-92260 Fontenay-aux-Roses, France.
[3]Nuclear Dynamics, CNRS UMR 3664, Institut Curie, PSL Research University, Sorbonne Université, Paris 75005, France.
[4]Institute Joliot, Commissariat à l'énergie Atomique (CEA), Direction de la Recherche Fondamentale (DRF), F91191 Gif-sur-Yvette, France
[5]Institute for Integrative Biology of the Cell (I2BC), CEA, CNRS, Univ. Paris-Sud, Université Paris-Saclay, 91198, Gif-sur-Yvette cedex, France
[6]Université Paris Cité, Inserm, CEA, Stabilité Génétique Cellules Souches et Radiations, CIGEx/iRCM/IBFJ, F-92260 Fontenay-aux-Roses, France.
[7]Université Paris-Saclay, Inserm, CEA, Stabilité Génétique Cellules Souches et Radiations, CIGEx/iRCM/IBFJ, F-92260 Fontenay-aux-Roses, France.
[8]Synchrotron SOLEIL, HelioBio group, l'Orme des Merisiers, Départementale 128, 91190 Saint-Aubin, France.

Supplementary Fig. 1 to 8

Supplementary Tables 1 to 4

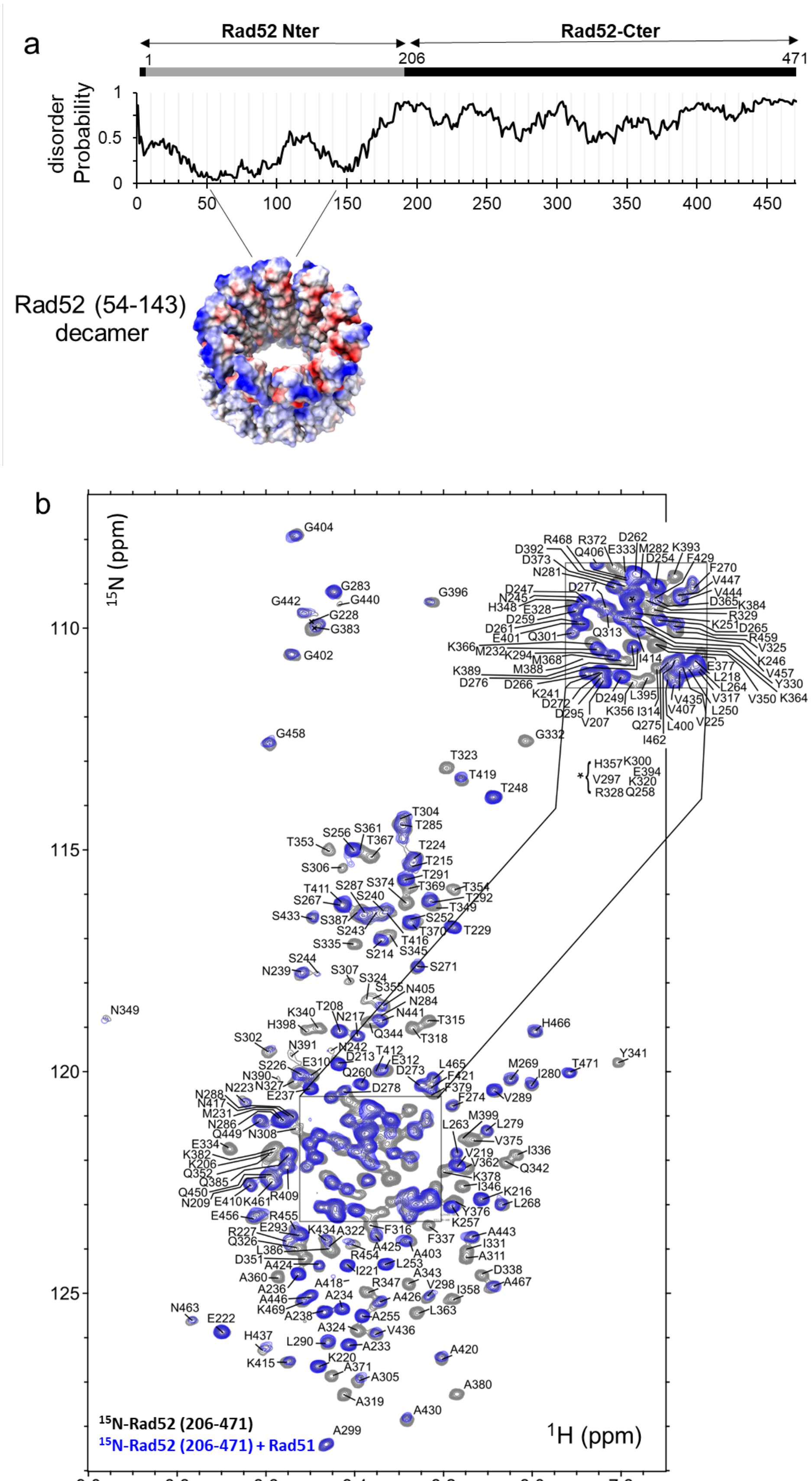

a
Rad52 Nter
Rad52-Cter
1
206
471
disorder Probability
1
0.5
0
0
50
100
150
200
250
300
350
400
450
Rad52 (54-143) decamer
b
15N (ppm)
110
115
120
125
1H (ppm)
9.0
8.8
8.6
8.4
8.2
8.0
7.8
15N-Rad52 (206-471)
15N-Rad52 (206-471) + Rad51

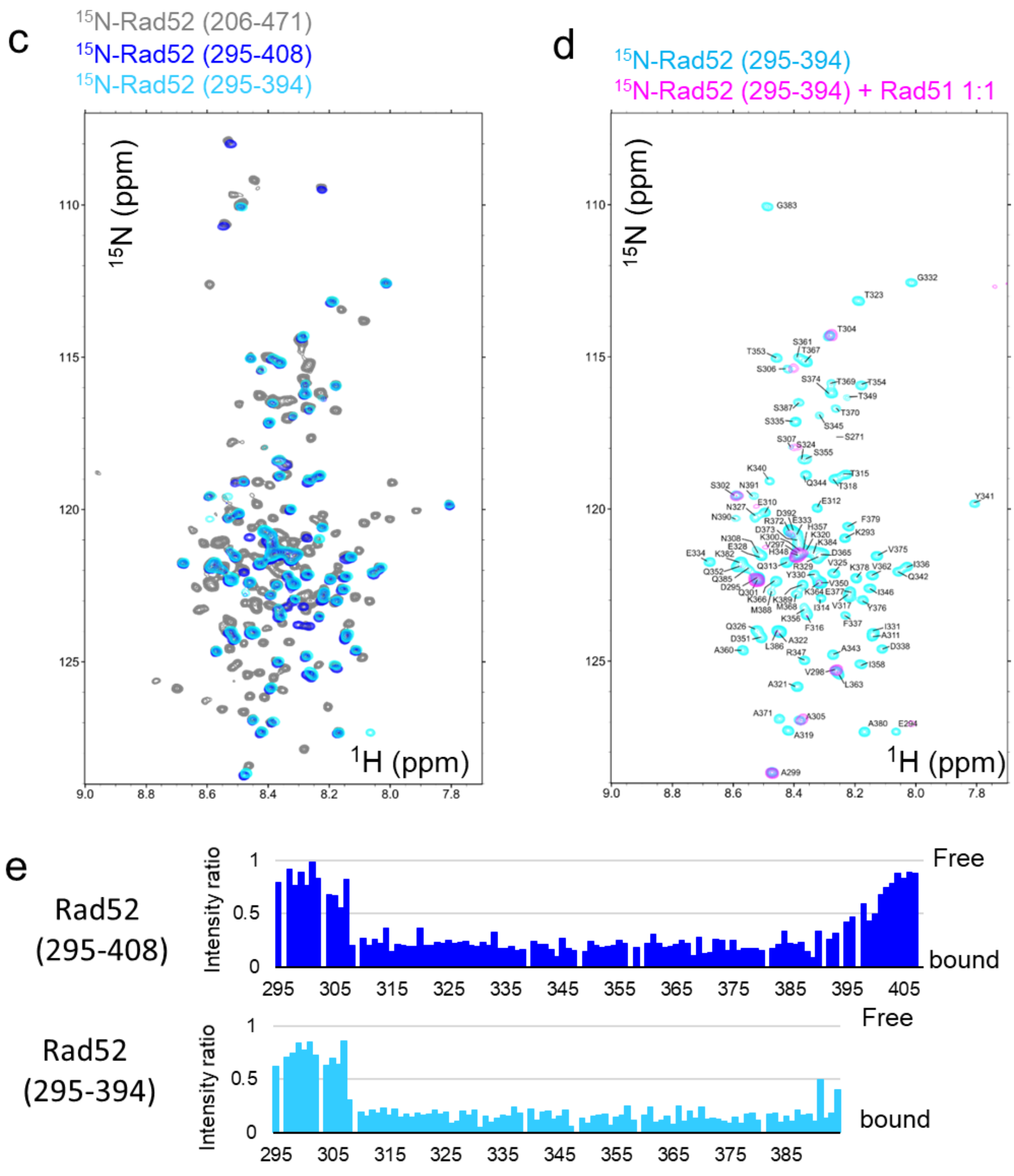

Supplementary Figure 1: Rad52 C-terminus is disordered and interacts with Rad51 with a central region of 85 residues. a General organisation of the N-terminal and C-terminal domains of Rad52 and the disorder probability for the full Rad52 sequence. The experimental decameric structure of the N-terminal region of yeast Rad52 (PDB:°8G3G) is shown as a surface with electrostatic colouring from blue for positive changes to red for negative charges **b** ^{1}H-^{15}N SOFAST-HMQC spectra of the uniformly ^{15}N labelled Rad52-Cter domain (206-471) alone in grey and, after addition of equimolar amount of unlabeled Rad51 in blue. The full assignment of residues is indicated. **c** ^{1}H-^{15}N SOFAST-HMQC spectra of uniformly ^{15}N labelled Rad52-Cter domain (206-471) in grey, (295-408) in blue and (295-394) in cyan. **d** ^{1}H-^{15}N SOFAST-HMQC spectra of the uniformly ^{15}N labelled Rad52-Cter domain (295-394) alone in cyan and, after addition of equimolar amount of unlabeled Rad51 in magenta. The assignment of residues is indicated. **e** Mapping of the interaction between Rad52-Cter domains (295-408 in the upper panel) or (295-394 in the lower pane) with Rad51, using the intensities ratio (I/I0), where I and I0 are the intensity of the signals ^{1}H-^{15}N SOFAST-HMQC spectra before and after addition of Rad51, respectively.

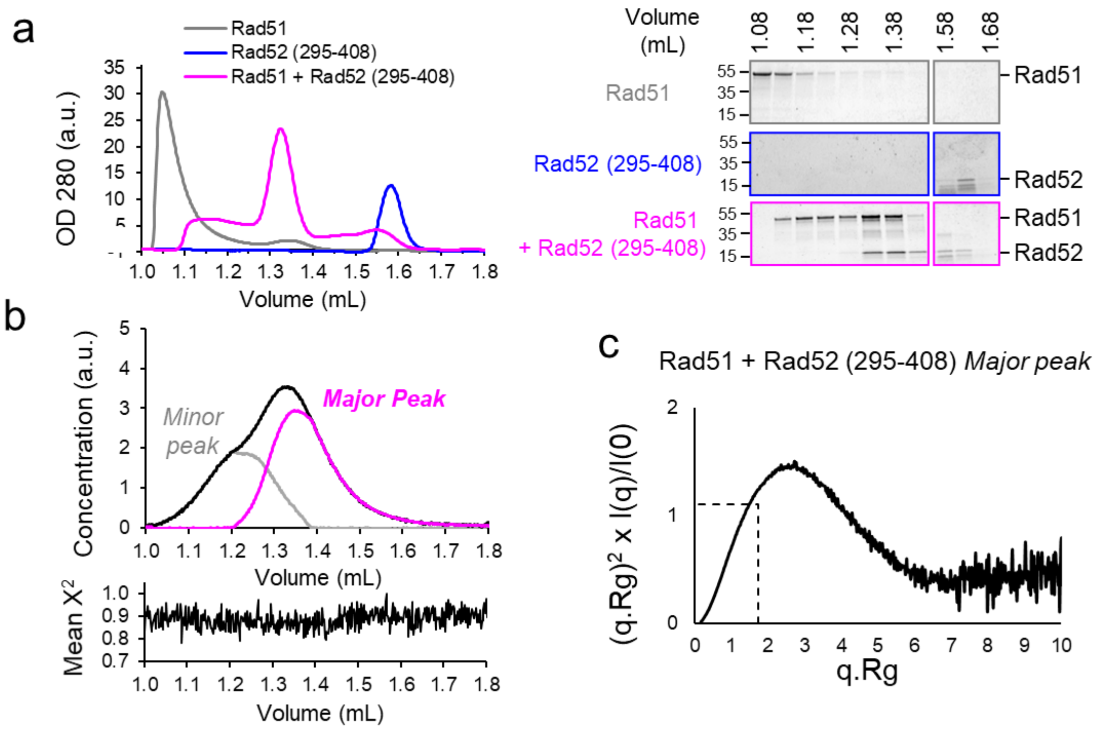

Supplementary Figure 2: Rad52 C-ter is disordered and interacts with Rad51 with central region of 85 residues. a Left panel: SEC profile analysis of Rad51, Rad52 (295-408) and Rad51+Rad52 (295-408) in a 1:1 ratio. Right panel: SDS-PAGE analysis of fraction from SEC profile in left panel revealed with coomassie blue. **b** Deconvolution of the SEC-SAXS curve using REGALS module [1] in BioXTAS RAW [2] upper panel, area-normalized concentration profiles for each component. With the major peak shown in magenta and the minor peak in grey. Lower panel: Mean χ^2 values of the fit of the deconvolution and the original data. **c** Dimensionless Kratky plot of the SAXS experiment of Rad51 + Rad52 (295-408) major peak after deconvolution. The expected maximum position for a fully globular protein is indicated by dashed black lines. The maximum of the experimental curve is shifted to higher values on both the y and x axes compared to this reference, indicating that the complex is extended with some flexible regions.

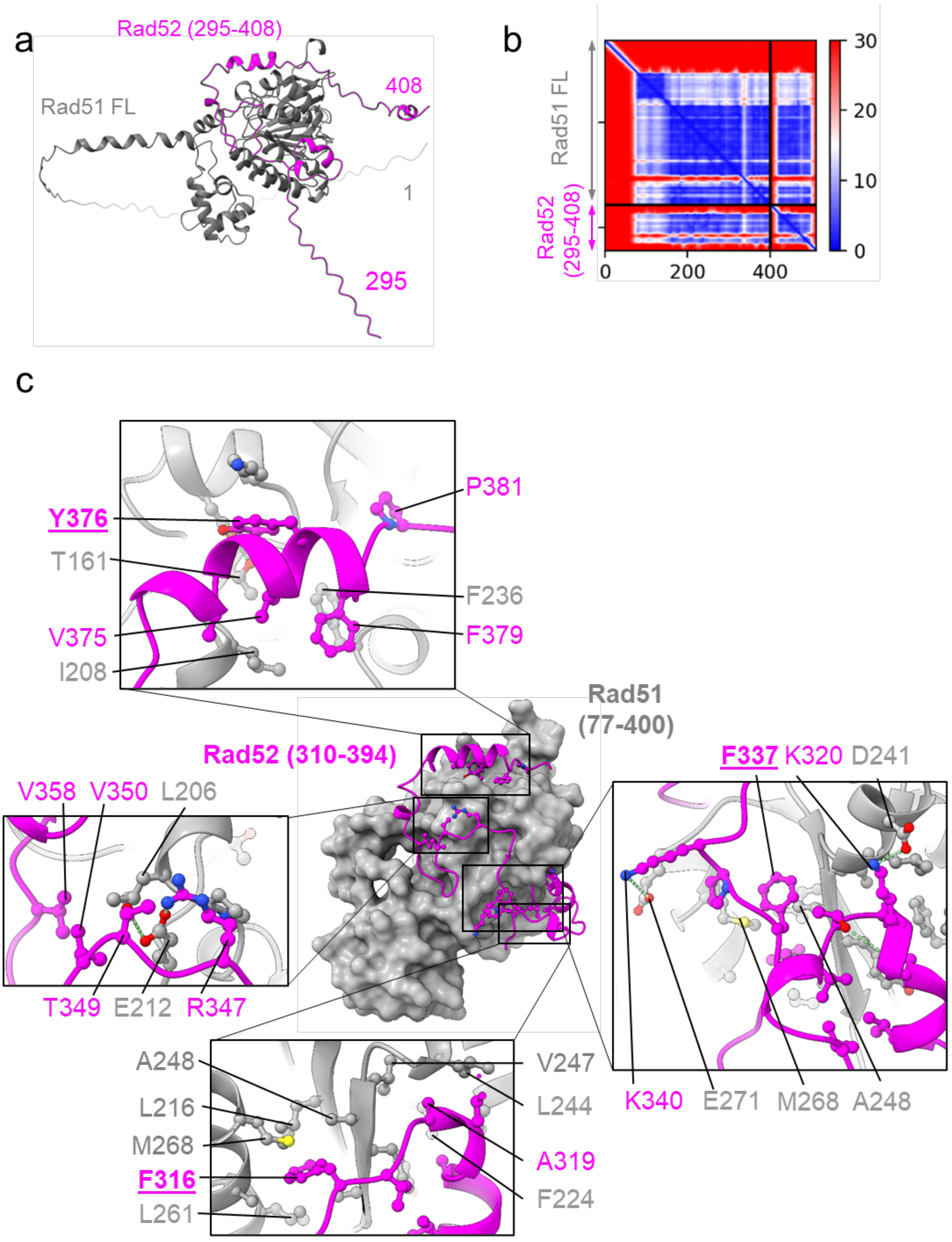

a
Rad52 (295-408)
Rad51 FL
408
1
295
b
Rad51 FL
Rad52 (295-408)
0
200
400
30
20
10
0
c
P381
Y376
T161
F236
V375
F379
I208
Rad51 (77-400)
Rad52 (310-394)
F337
K320
D241
V358
V350
L206
T349
E212
R347
K340
E271
M268
A248
A248
V247
L216
L244
M268
A319
F316
F224
L261

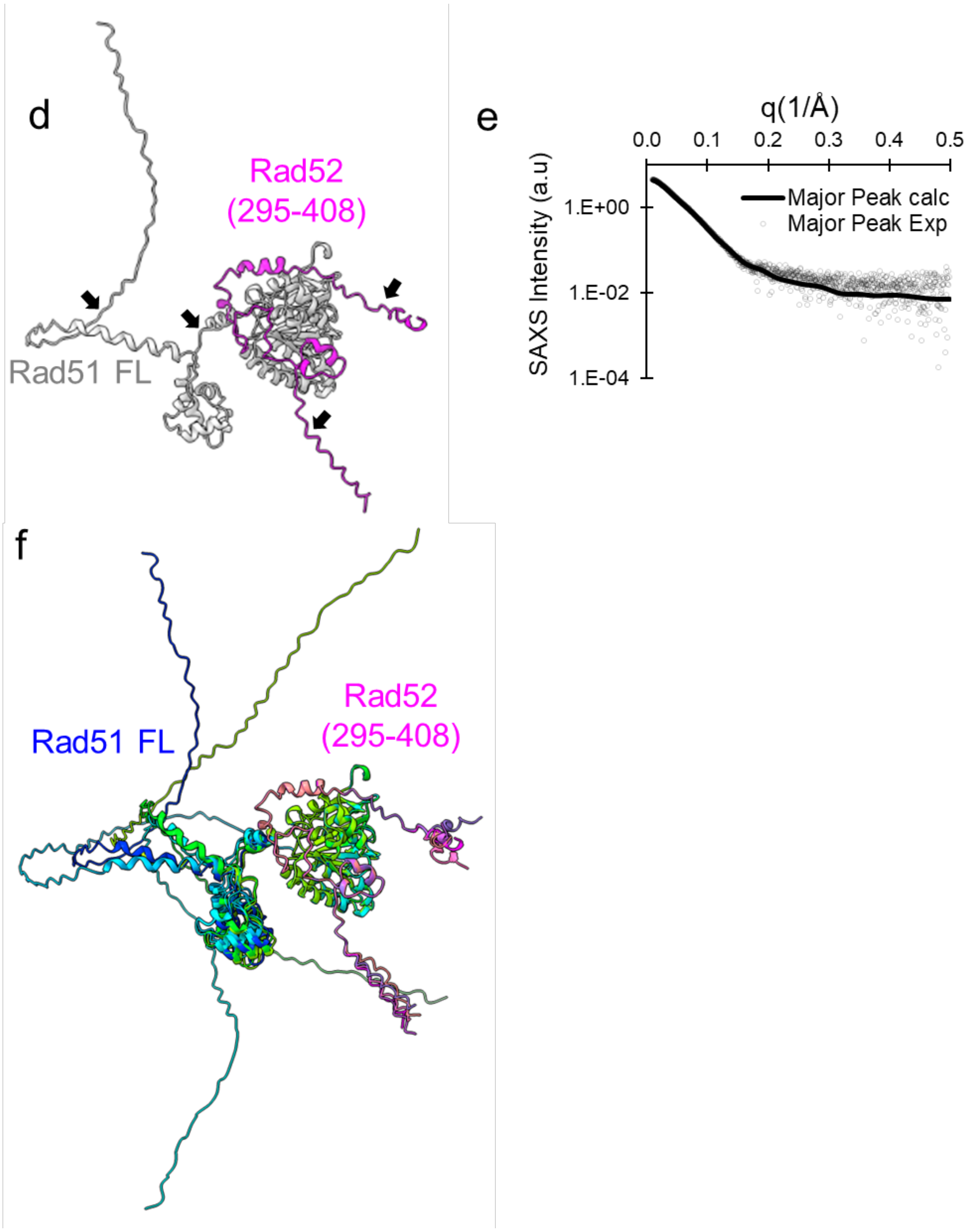
d
Rad52
(295-408)
Rad51 FL
e
q(1/Å)
0.0
0.1
0.2
0.3
0.4
0.5
SAXS Intensity (a.u)
1.E+00
1.E-02
1.E-04
Major Peak calc
Major Peak Exp
f
Rad51 FL
Rad52
(295-408)

g

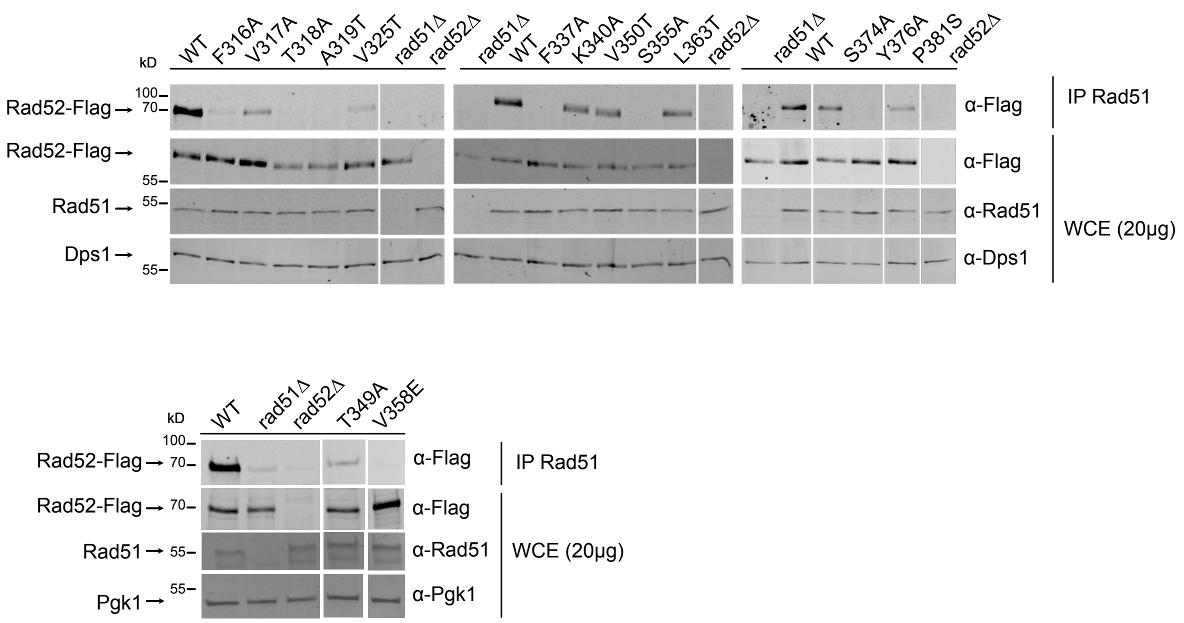

h

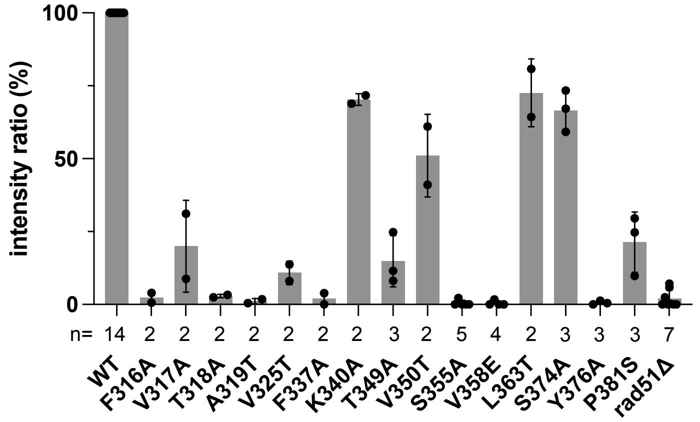

Supplementary Figure 3: Rad52 C-ter is disordered and interacts with Rad51 with central region of 85 residues. a AF2 model of the complex between Rad51 + Rad52 (295-408). Both proteins are shown as cartoons, with Rad51 in grey and Rad52 in magenta. **b** Predicted Alignment Error plot (PAE) calculated by AlphaFold2 for the best model of Rad51+Rad52 (295-408) presented in panel A. **c** Central panel: AF2 model of the complex between Rad51 (77-400) + Rad52 (310-394). Rad51 is displayed as a grey surface, Rad52 as a magenta cartoon, with key residues in contact with Rad51 side chains in ball and sticks. Four boxes highlight zoomed-in regions of the interface, showing the side chains of key interacting residues, with the same color. Rad51 residues in direct contact with Rad52 and in particular with the three anchor residues—F316, F337, and Y376—are labeled. A complete list of intermolecular contacts is given in Supplementary Table 1. **d** Ribbon representation of the best model generated by the Dadimodo software [3]. Both proteins are shown as cartoons, with Rad51 in grey and Rad52 in magenta. The position of the last residue of the N-terminal tail of Rad51, and the hinge between the N-terminal four-helix domain and the C-terminal domain are indicated with black arrows. the positions of residues 310 and 394, which delimit the two extremities considered flexible in Rad52, are also indicated with black arrows. **e** Experimental and fitted SAXS profile intensity (I) as a function of the momentum

transfer (q) for Rad51 + Rad52 (295-408) major peak after deconvolution (black circles) and for the best model generated by the Dadimodo Software (continuous black line) shown in panel D. **f** Overlay of the 5 best models generated by the Dadimodo software. A color gradient from blue to green is used for Rad51, and shades of pink, purple, and orange are used for Rad52. **g** Co-IP experiments between Rad52 mutants and Rad51. Rad51 was immunoprecipitated with a poly-clonal anti-Rad51 antibody (αRad51). The presence of Rad51 in the immunoprecipitated fraction (IP) cannot be detected because it migrates at the same level as the anti-Rad51 IgG used for the immunoprecipitation. However, the absence of Rad52-FLAG in the *rad51*Δ immunoprecipitate confirmed that the Rad52-FLAG signal observed is related to the Rad52–Rad51 interaction. Western blot analysis of Rad52-FLAG, Rad51 and Dps1 or Pgk1 in whole cell extracts (WCE) reveals that the amount of proteins is comparable in each mutant strains. Four representative experiments are shown (horizontal bars), each with their relative WT, *rad51*Δ and *rad52*Δ controls. White lanes indicate cut off of experiments not related to this study. Position of molecular weight markers are indicated (in kDa). Uncropped blots are provided in the Source Data file.**h** Intensity ratio between mutant and WT Rad52 co-immunoprecipitated with Rad51. Data are presented as the mean ± SEM. n indicates the number of independent experiments.

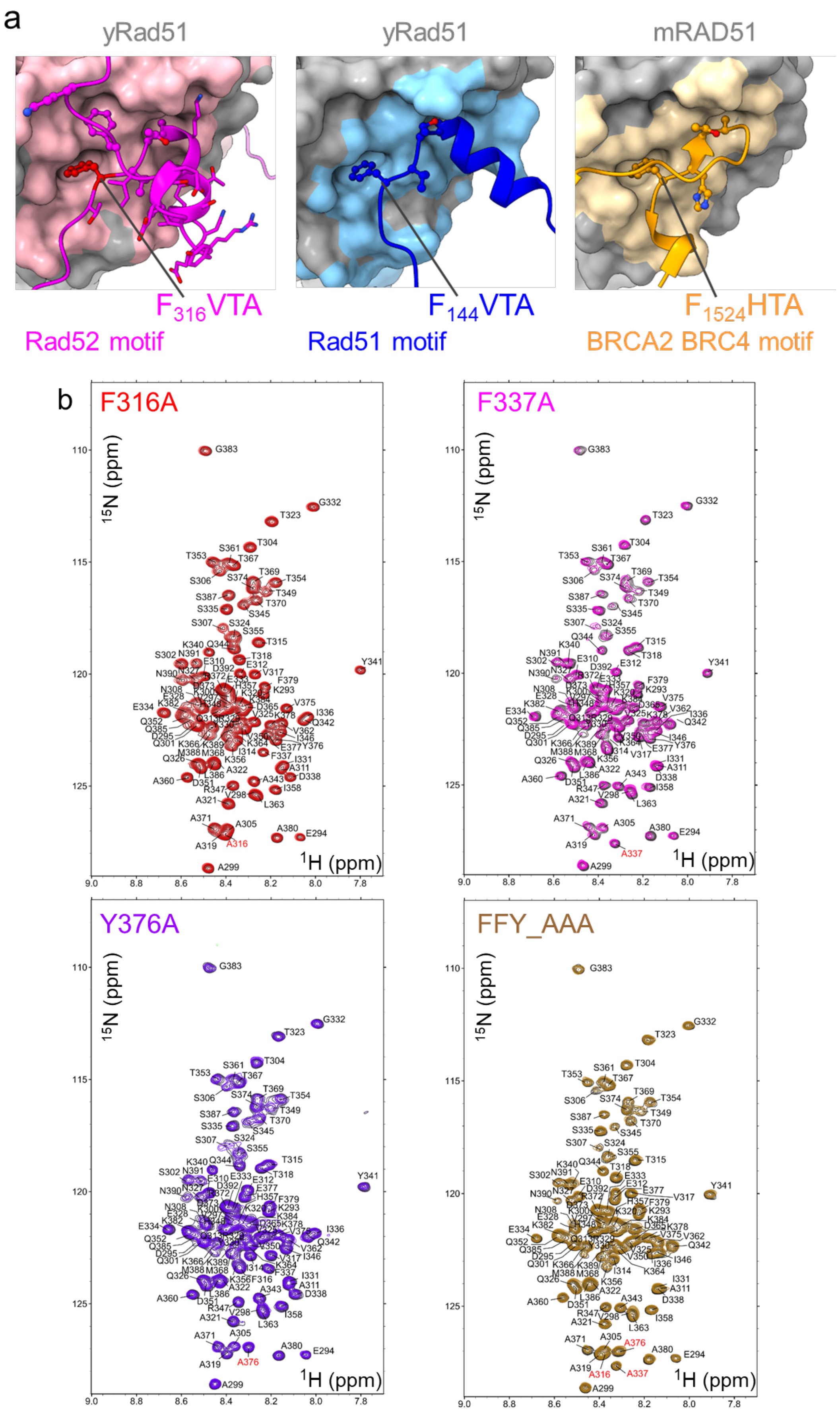
a
yRad51
yRad51
mRAD51
F316VTA
Rad52 motif
F144VTA
Rad51 motif
F1524HTA
BRCA2 BRC4 motif
b
F316A
F337A
Y376A
FFY_AAA
15N (ppm)
1H (ppm)

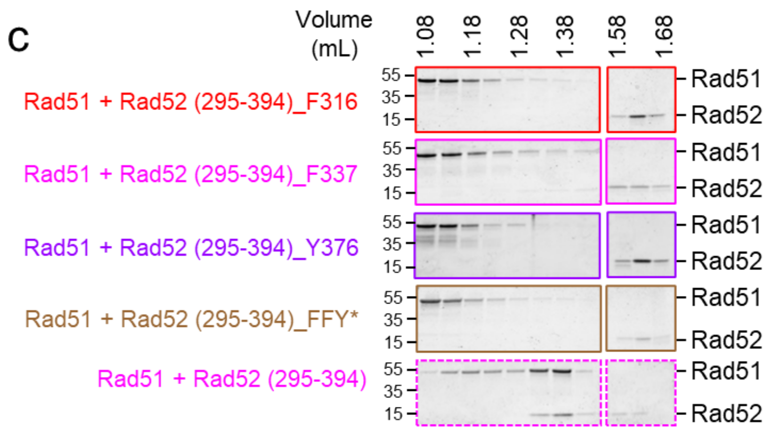

Supplementary Figure 4: Rad52 (310–394) competes with Rad51 oligomers through the cooperative binding of several anchors. a Zoomed-in view of the region of Rad51 interacting with FxxA motif. Left panel AF2 model of the complex between Rad51 and Rad52. Rad51 is displayed as a surface, with residues in contact with Rad52 colored pink and other residues in grey. Rad52 is shown as a magenta cartoon, with visible side chains. Middle panel, interaction of two Rad51 monomers assembled in a multimer (PDB code 1SZP). One Rad51 monomer is shown as a surface, with residues in contact with the second Rad51 colored blue and other residues in grey. The second monomer, containing the FVTA motif, is displayed as a blue cartoon, with visible side chains for the FVTA motif. Right panel Structure of mouse RAD51 interacting with the FHTA BRC4 motif of BRCA2 (PDB code 1N0W). Rad51 is shown as a surface, with residues in contact with BRCA2 colored orange. BRCA2 is represented as an orange cartoon, with visible side chains for the FHTA motif. **b** ^{1}H-^{15}N SOFAST-HMQC spectra of the four Rad52-Cter domain (295-394) mutants, uniformly ^{15}N labelled, shown alone in black and after addition of an equimolar amount of unlabeled Rad51 in red, magenta, purple, and brown for F316A, F337A, Y376A and the triple mutant FFY respectively. Residues assignments are indicated. **c** SDS-PAGE analysis of fractions from SEC profiles in **Figure 4D,** stained with coomassie blue.

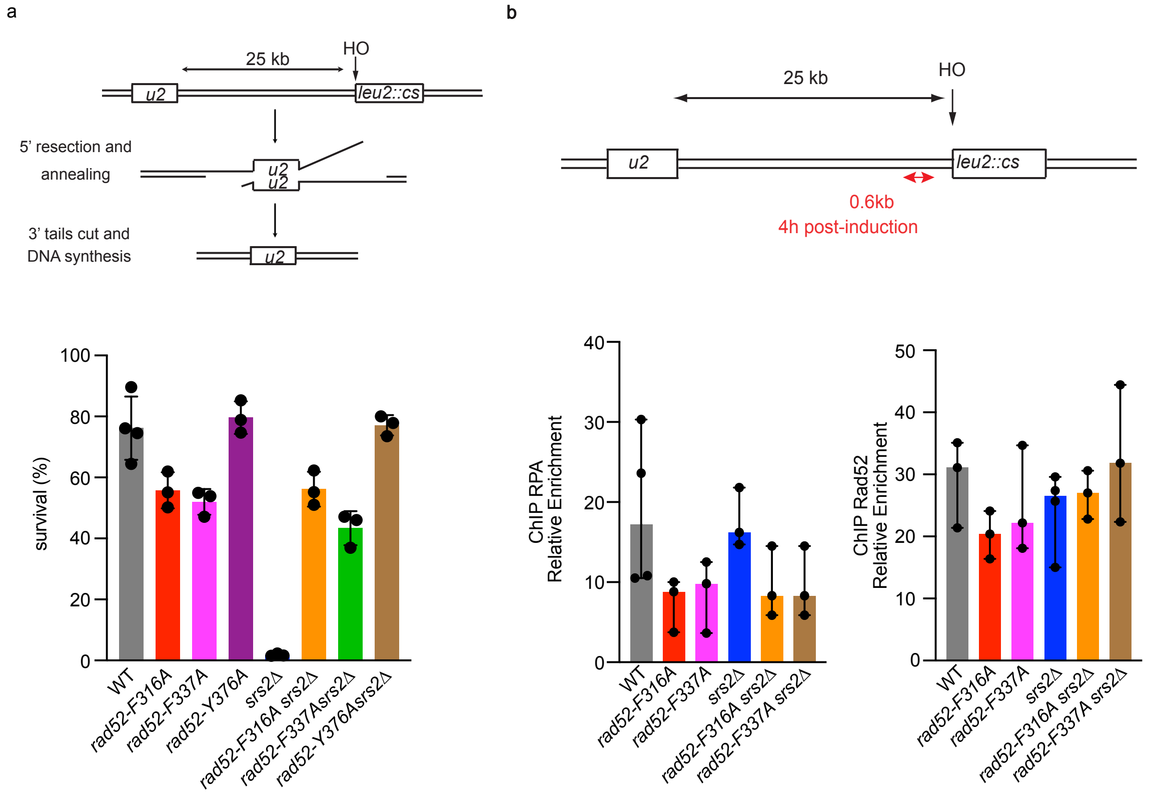

Supplementary Figure 5: Anchor 1 and 2 are more important for Rad51 filament formation than Anchor 3. **a:** Upper panel: schematic of the different steps of an HO-induced DSB repair by SSA. Cell survival after HO-induced DSB formation in a SSA repair system. Lower panel: data are presented as the mean ± SEM. n=3 independent experiments except for WT and rad52-F337, n=4. Already published data obtained with *rad52-Y376A* are shown for reference [4] (CC BY 4.0: https://creativecommons.org/licenses/by/4.0/). **b** Upper panel: schematic of the HO-induced SSA repair system is shown. Lower panel: ChIP was used to assess RPA and Rad52 relative enrichment at 0.6 kb from the DSB site (red) 4 hours after HO induction. Data are presented as the median and the error bars represent the minimum and maximum values. None of the results are statistically significant (two-tailed unpaired t-test). n=3 independent experiments except for WT and *srs2*Δ, n=4. Source data are provided in the Source Data file.

**Impact of *rad52* mutations**			
Conditions	p-value filament length	p-value filament intensity	p-value Foci intensity
WT vs F337A	n.a.	n.a.	4.07 e-6 ***
WT vs Y376A	0.569	0.402	1.95 e-5 ***
WT vs F316A	n.a.	n.a.	1.63 e-6 ***

**Impact of *SRS2* deletion on WT and *rad52* mutant strains**			
Conditions	p-value filament length	p-value filament intensity	p-value Foci intensity
WT vs srs2Δ	0.590	1.02 e-8 ***	0.0171
F337A vs F337A, srs2Δ	n.a.	n.a.	2.10 e-12 ***
Y376A vs Y376A , srs2Δ	0.865	0.164	5.89 e-9 ***
F316A vs F316A, srs2Δ	n.a.	n.a.	6.08 e-16 ***

**Impact of *rad52* mutations on *SRS2* deleted strains**			
Conditions	p-value filament length	p-value filament intensity	p-value Foci intensity
srs2Δ vs F337A, srs2Δ	0.0363*	7.28 e-12 ***	4.20 e-5 ***
srs2vs Y376A , srs2Δ	0.0150*	8.00 e-13 ***	0.0101
srs2 vs F316A, srs2Δ	0.0336*	7.14 e-5 ***	7.05 e-7 ***

***srs2Δ rad52 mutants vs* WT**			
Conditions	p-value filament length	p-value filament intensity	p-value Foci intensity
WT vs F337A, srs2Δ	3.41 e-6 ***	0.210	0.275
WT vs Y376A , srs2Δ	4.56 e-7 ***	1	1
WT vs F316A, srs2Δ	0.654	5.30 e-8 ***	4.56 e-7 ***

Supplementary Figure 6: Statistical test corresponding to Figure 6.
Logistic regression with binomial distribution (see materials and methods; ***P < 0.001, **P < 0.01, *P < 0.05).

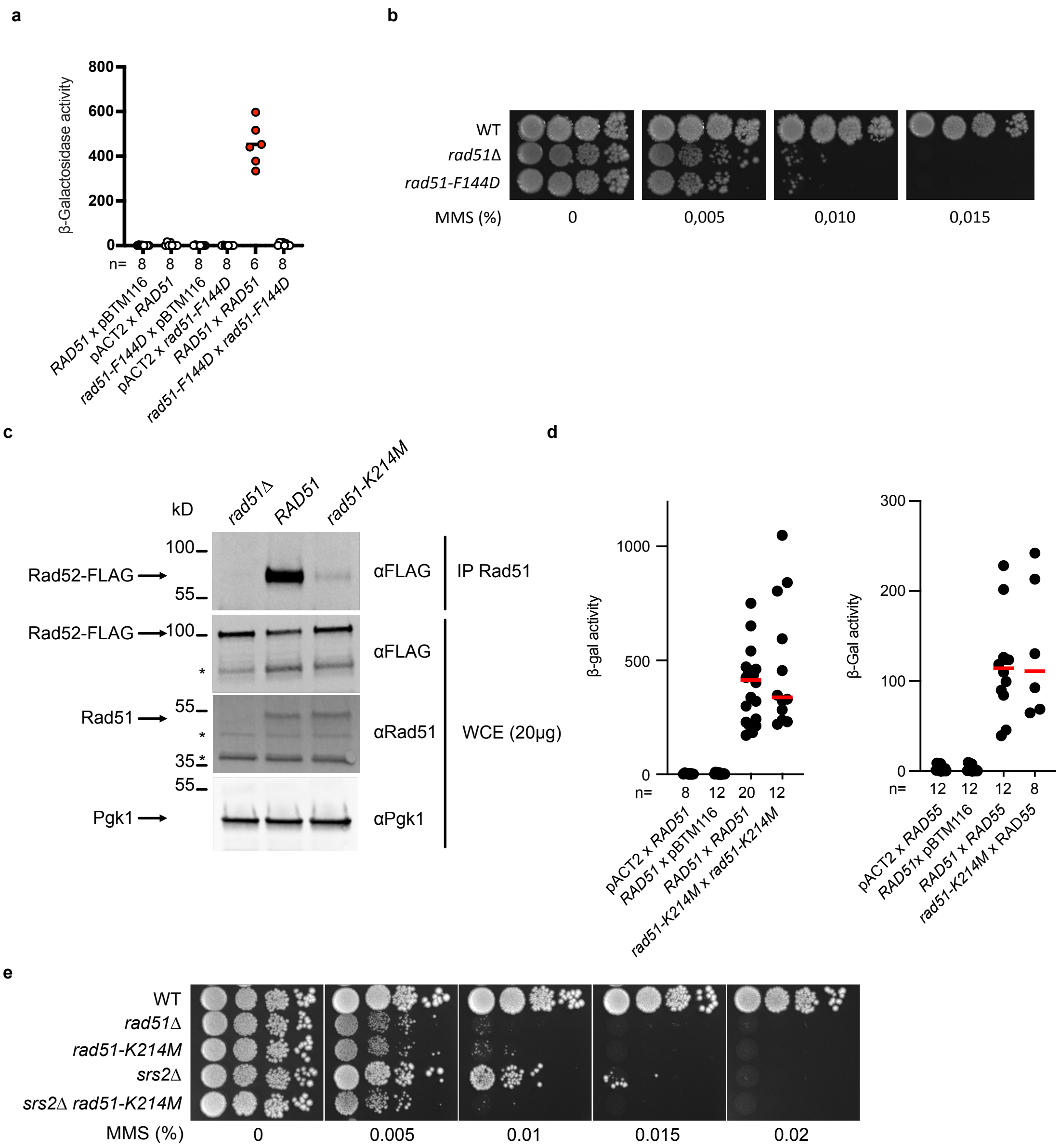

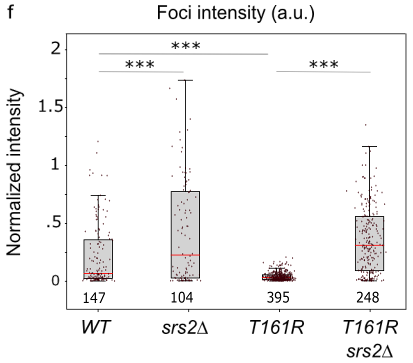

g

Conditions	p-values Filament Intensity	p-values Filament length	p-values Foci Intensity
wt vs srs2	9.14 e-12 ***	0.847	5.93 e-5 ***
wt vs T161R	1.26 e-3 *	0.847	3.12 e-11 ***
wt vs srs2 T161R	3.38 e-3 *	1.79 e-8 ***	3.47 e-5 ***
T161R vs T161R srs2	8.85 e-4 **	0.847	9.13 e-21 ***
srs2 vs T161R srs2	4.31 e-5 ***	8.08 e-9 ***	0.0673

Supplementary Figure 7. Rad51-F144D is defective in Rad51-Rad51 interaction and Rad51-K214M is highly defective in HR. a Y2H analysis of Rad51-F144D interaction with itself. Semi-quantitative measurement of β-galactosidase activity in independent colonies issued from co-transformation of plasmids bearing the LexA-activating domain (pACT2) and the GAL4 binding domain (pBTM) either empty or bearing fusion proteins. Note that in addition of F144D, *RAD51* is also mutated for E221K, which suppresses the toxicity of Rad51 overexpression (see Materials and Methods). n indicates the number of colonies tested. **b** Serial 10-fold dilutions of haploid strains with

the indicated genotypes were spotted onto rich medium (YPD) containing different MMS concentrations. **c** Co-IP experiments showing the loss of interaction between Rad51-K214M and Rad52. Rad51 was immunoprecipitated with a poly-clonal anti-Rad51 antibody (αRad51). The presence of Rad51 in the immunoprecipitated fraction (IP) cannot be detected because it migrates at the same level as the anti-Rad51 IgG used for the immunoprecipitation. However, the absence of Rad52-FLAG in the *rad51*Δ immunoprecipitate confirmed that the Rad52-FLAG signal observed is related to the Rad52–Rad51 interaction. Western blot analysis of Rad52-FLAG, Rad51 and Pgk1 in whole cell extracts (WCE) reveals that the amount of proteins is comparable in each mutant strains. Position of molecular weight markers are indicated (in kDa).* Unspecific bands. Uncropped blots are provided in the Source Data file. **d** Y2H analysis of Rad51-K214M interaction with itself and with Rad55-Rad57. Semi-quantitative measurement of β-galactosidase activity in independent colonies issued from co-transformation of plasmids bearing the LexA-activating domain (pACT2) and the GAL4 binding domain (pBTM) either empty or bearing fusion proteins. Note that in addition of K214M, *RAD51* is also mutated for E221K, which suppresses the toxicity of Rad51 overexpression (see Materials and Methods). n indicates the number of colonies tested. **e** Serial 10-fold dilutions of haploid strains with the indicated genotypes were spotted onto rich medium (YPD) containing different MMS concentrations. **f** Comparison of Rad51 foci intensities in WT and mutant strains as indicated. The number of structures analyzed is indicated below each box. Intensities are normalized to the mean intensity of WT Rad51 filaments. On each box, the central mark indicates the median, and the bottom and top edges of the box indicate the 25th and 75th percentiles, respectively. The whiskers extent corresponds to the adjacent value, which is the most extreme data value not considered an outlier (above 75th percentile + 1.5 times interquartile range or below 25th percentile - 1.5 times interquartile range). Exact p-values for all comparisons are presented in g. **g** Statistical test corresponding to Figure 7F. logistic regression with binomial distribution, t-statistic on coefficients corrected for multiple comparison with the False Discovery Rate (see materials and methods).

a

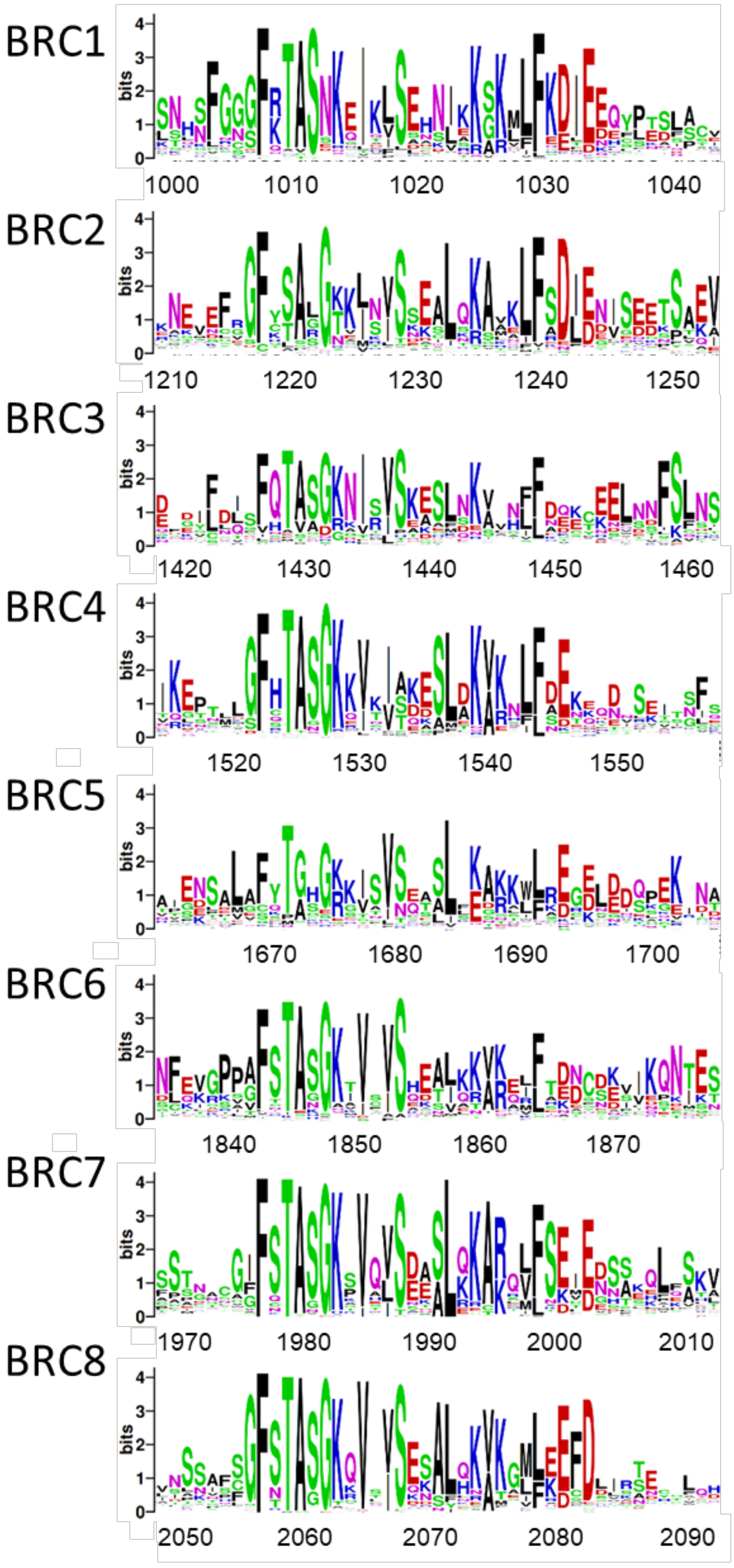
BRC1
1000 1010 1020 1030 1040
BRC2
1210 1220 1230 1240 1250
BRC3
1420 1430 1440 1450 1460
BRC4
1520 1530 1540 1550
BRC5
1670 1680 1690 1700
BRC6
1840 1850 1860 1870
BRC7
1970 1980 1990 2000 2010
BRC8
2050 2060 2070 2080 2090
bits

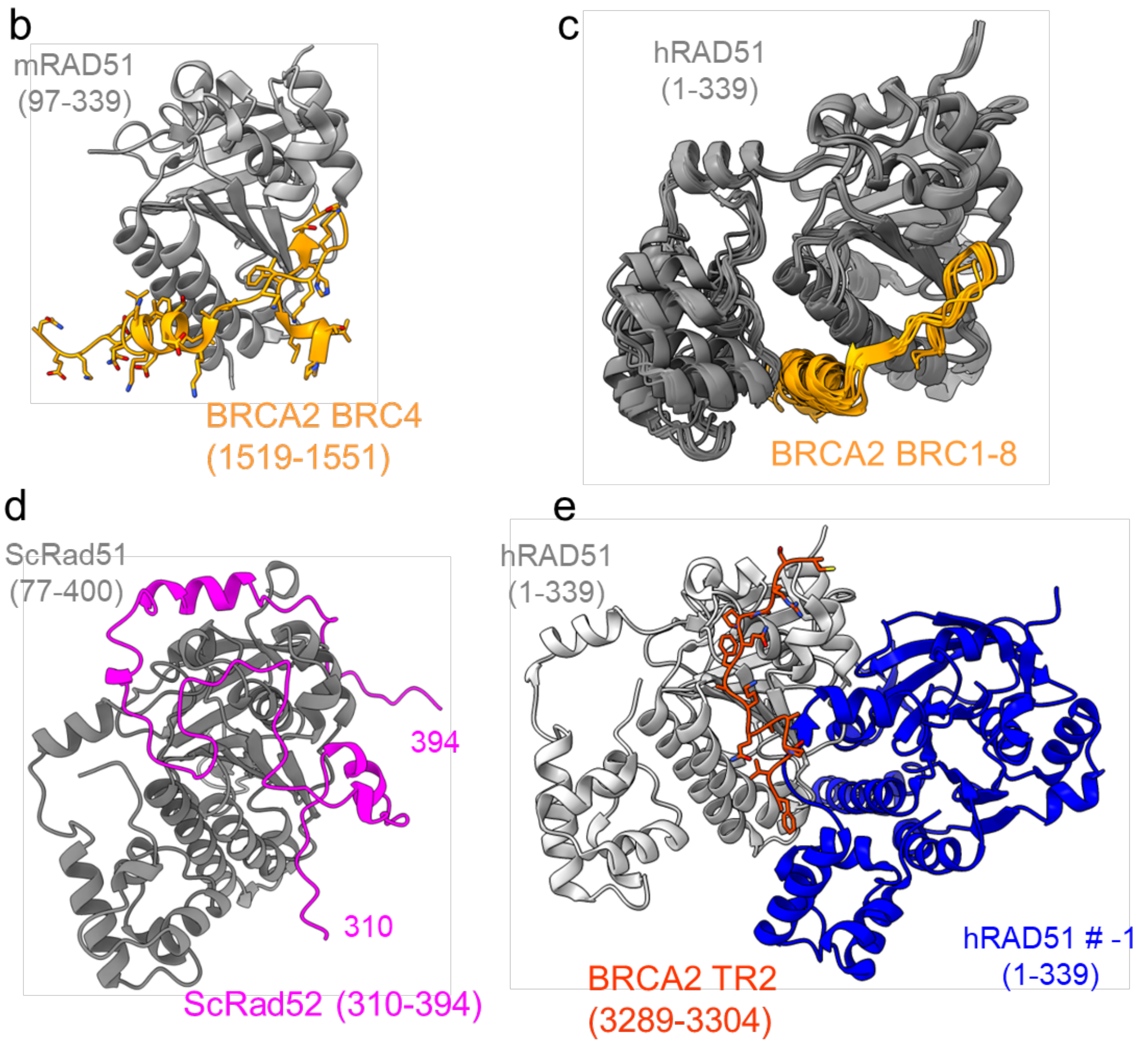

Supplementary Figure 8. The BRC Rad51-binding regions of BRCA2 include an FxxA motif extended by a helical segment that interacts with a Rad51 surface distinct from that targeted by Rad52. a Sequence Logo of the eight BRC motifs of BRCA2. **b** Crystal structure of mouse RAD51 in complex with the BRC4 motif of BRCA2 (PDB code: 1N0W). RAD51 is shown in grey as cartoon, BRC4 is represented in orange as cartoon with visible side chains. A close-up view of the FxxA motif is shown in Supplementary Fig. 4A. **c** Overlay of AlphaFold2-predicted structures of human RAD51 (grey cartoon) in complex with each of the eight BRC motifs of BRCA2 (orange cartoons). **d** AlphaFold2-predicted structures of yeast Rad51 (grey cartoon) in complex with the Rad52 C-terminal region (magenta cartoon) presented in Fig. 3 and 4, Supplementary Fig. 3 and 4) in the same orientation as panels B, C and E to facilitate comparison of binding modes. **e** Two monomers of the cryoEM structure of human RAD51 bound to the TR2 motif of BRCA2 (3289-3303) (PDB code: 8PBC).

Supplementary Table 1. List of contacts between Rad52 and Rad51 (sc = side chain, bb= backbone) corresponding to Supplementary Fig. 3C

Rad52		Rad51		Interaction type
**E**	312	R	260	Charged Contact
**I**	314	R	260	Hydrophobic Contact
**I**	314	L	261	Hydrophobic Contact
**I**	314	A	264	Hydrophobic Contact
**F**	316	L	216	Hydrophobic Contact
**F**	316	I	218	Hydrophobic Contact
**F**	316	A	248	Hydrophobic Contact
**F**	316	A	250	Hydrophobic Contact
**F**	316	L	261	Hydrophobic Contact
**F**	316	A	264	Hydrophobic Contact
**F**	316	M	268	Hydrophobic Contact
**V**	317	Y	249	Hydrophobic Contact
**V**	317	Y	249	Hydrogen Bond bb-bb
**A**	319	F	224	Hydrophobic Contact
**A**	319	L	229	Hydrophobic Contact
**A**	319	L	244	Hydrophobic Contact
**A**	319	L	244	Hydrogen Bond bb-bb
**A**	319	V	247	Hydrophobic Contact
**A**	319	V	247	Hydrogen Bond bb-bb
**K**	320	D	241	Hydrogen Bond sc-sc
**K**	320	D	241	Salt Bridge
**K**	320	L	244	Hydrophobic Contact
**A**	322	F	224	Hydrophobic Contact
**A**	322	P	226	Hydrophobic Contact
**A**	322	Y	249	Hydrophobic Contact
**V**	325	Y	249	Hydrophobic Contact
**V**	325	R	251	Hydrophobic Contact
**Q**	326	D	219	Hydrogen Bond sc-sc
**Q**	326	F	224	Hydrogen Bond sc-bb
**F**	337	K	214	Hydrophobic Contact
**F**	337	A	248	Hydrophobic Contact
**F**	337	M	268	Hydrophobic Contact
**F**	337	F	274	Hydrophobic Contact
**P**	339	K	214	Hydrogen Bond bb-sc
**P**	339	M	268	Hydrophobic Contact
**P**	339	S	272	Hydrogen Bond bb-sc
**K**	340	E	271	Charged Contact
**Y**	341	K	214	Hydrogen Bond bb-sc
**Y**	341	K	214	Cation-Pi
**Y**	341	D	242	Hydrogen Bond sc-sc
**Y**	341	N	245	Hydrogen Bond sc-sc
**Q**	344	D	242	Hydrogen Bond bb-sc
**S**	345	D	242	Hydrogen Bond bb-sc

Rad52		Rad51		Interaction type
**I**	346	L	238	Hydrophobic Contact
**R**	347	P	205	Hydrophobic Contact
**R**	347	D	207	Hydrogen Bond sc-sc
**R**	347	D	207	Salt Bridge
**R**	347	E	212	Charged Contact
**H**	348	E	212	Charged Contact
**T**	349	E	212	Hydrogen Bond bb-sc
**T**	349	E	212	Hydrogen Bond sc-sc
**V**	350	L	206	Hydrophobic Contact
**V**	350	R	273	Hydrophobic Contact
**Q**	352	E	212	Hydrogen Bond sc-bb
**Q**	352	R	273	Hydrogen Bond bb-bb
**S**	355	M	269	Hydrogen Bond bb-bb
**S**	355	M	269	Hydrogen Bond sc-bb
**S**	355	S	272	Hydrogen Bond sc-bb
**S**	355	F	274	Hydrogen Bond sc-bb
**K**	356	S	79	Hydrogen Bond sc-sc
**K**	356	F	80	Hydrophobic Contact
**K**	356	R	273	Hydrogen Bond bb-sc
**H**	357	D	315	Hydrogen Bond sc-bb
**I**	358	I	158	Hydrophobic Contact
**I**	358	L	206	Hydrophobic Contact
**A**	360	E	156	Hydrogen Bond bb-bb
**L**	363	I	158	Hydrophobic Contact
**L**	363	L	206	Hydrophobic Contact
**K**	366	D	207	Charged Contact
**D**	373	K	165	Hydrogen Bond sc-sc
**D**	373	K	165	Salt Bridge
**V**	375	I	208	Hydrophobic Contact
**Y**	376	T	161	Hydrogen Bond sc-sc
**Y**	376	K	165	Hydrophobic Contact
**Y**	376	D	168	Hydrogen Bond sc-sc
**F**	379	I	208	Hydrophobic Contact
**F**	379	R	235	Hydrogen Bond bb-sc
**F**	379	R	235	Cation-Pi
**F**	379	F	236	Hydrophobic Contact
**P**	381	Y	388	Hydrophobic Contact
**P**	381	G	393	Hydrophobic Contact
**K**	382	D	390	Hydrogen Bond bb-sc
**K**	384	E	389	Hydrogen Bond bb-bb
**Q**	385	D	399	Hydrogen Bond sc-sc
**L**	386	I	387	Hydrophobic Contact
**L**	386	I	387	Hydrogen Bond bb-bb

Supplementary Table 2. List of contacts between Rad51 and Rad51 in a multimer, PDB code 1SZP (sc = side chain, bb= backbone) corresponding to Figure 4A and Supplementary Figure 4A

Rad51		Rad51		Interaction type
**A**	111	N	254	Hydrogen Bond bb-sc
**Y**	112	Y	253	Hydrophobic Contact
**M**	142	Y	253	Hydrophobic Contact
**F**	144	L	216	Hydrophobic Contact
**F**	144	I	218	Hydrophobic Contact
**F**	144	A	248	Hydrophobic Contact
**F**	144	A	250	Hydrophobic Contact
**F**	144	L	261	Hydrophobic Contact
**F**	144	A	265	Hydrophobic Contact
**F**	144	M	268	Hydrophobic Contact
**V**	145	Y	249	Hydrophobic Contact
**V**	145	Y	249	Hydrogen Bond bb-bb
**A**	147	V	247	Hydrophobic Contact
**A**	147	V	247	Hydrogen Bond bb-bb
**A**	148	L	244	Hydrophobic Contact
**F**	150	P	226	Hydrophobic Contact
**F**	150	Y	249	Hydrophobic Contact
**E**	176	R	225	Hydrogen Bond sc-sc
**E**	176	R	225	Salt Bridge
**K**	305	T	288	Hydrophobic Contact
**R**	312	D	289	Charged Contact
**D**	315	R	251	Hydrogen Bond sc-sc
**D**	315	R	251	Salt Bridge
**I**	349	F	187	Hydrophobic Contact
**I**	349	V	328	Hydrophobic Contact
**A**	351	R	188	Hydrophobic Contact
**H**	352	E	221	Charged Contact
**P**	376	R	225	Hydrophobic Contact
**C**	377	V	227	Hydrophobic Contact

Supplementary Table 3: Experimental information and modelling of SAXS data

**Sample details**	**Rad51 + Rad52 (295-308)**
Organism	*S. cerevisiae*
Source (catalogue No. or reference)	Recombinant proteins (See Methods)
UniProt sequence ID (residues in construct)	P25454(FL 1-400) P06778 (295-408)
Extinction coefficient [A_{280}, 0.1%(*w/v*)]	0.295
M from chemical composition (Da)	55 584
SEC–SAXS column	S200 3.2/300 Increase
Loading concentration (mg ml^{-1})	1.3
Injection volume (µl)	50
Flow rate (ml min^{-1})	0.05
Solvent (solvent blanks taken from SEC flowthrough prior to elution of protein)	Tris 20 mM, NaCl 100 mM, pH 8
**SAXS data-collection parameters.**	
Instrument/data processing	BioSAXS on the SWING beamline at Synchrotron SOLEIL(Thureau et al. 2021)
Wavelength (Å)	1.0332
Beam size (µm)	500x200
Camera length (m)	2.00
q measurement range ($Å^{-1}$); $q = 4\pi\sin(\theta)/\lambda$ (2θ: scattering angel & λ the x-ray wavelength)	0.00365–0.5538
Absolute scaling method	Comparison with scattering from 1 mm pure H_2O
Normalization	To transmitted intensity by beam-stop counter
Monitoring for radiation damage	data frame-by-frame comparison
Exposure time	Continuous 1 s data-frame measurements of SEC elution
Sample configuration	SEC–SAXS with thermalized quartz capillary (ID 1.5mm)
Sample temperature (°C)	20
**Software employed for SAXS data reduction, analysis and interpretation**	
SAXS data reduction	I(q) versus q, buffer subtraction & frames selection using Foxtrot 3.10 [a]
Extinction coefficient estimate	ProtParam [5]
Basic analyses: Guinier, *P(r)*, MW	PRIMUSqt from ATSAS 3.2.1 [6]
Atomic structure modelling	Dadimodo [3] (https://dadimodo.synchrotron-soleil.fr/)
Missing sequence modelling	AlphaFold2 [7]
Three-dimensional graphic model representations	PyMOL v.2.00
**Structural parameters**	
Guinier analysis	
I(0) (cm^{-1})	4.75 ± 5E-3
R_g (Å)	39.36 ± 0.06
q_{min} ($Å^{-1}$)	0.0114
qR_g max (q min = 0.0066 $Å^{-1}$)	1.33
Coefficient of correlation, R^2	0.998
M from Vc (ratio to predicted)	68 300 (0.81)
P(r) analysis	
I(0) (cm^{-1})	4.86 ± 9E-3
R_g (Å)	43.0 ± 0.19
d_{max} (Å)	170
q range ($Å^{-1}$)	0.011 to 0.554
total estimate from GNOM	0.724
**Atomistic modelling.**	
Crystal structures	
q range for all modelling	0.01003–0.49998
PepsiSAXS (r0 fixed)	
χ^2	2.69
Vol ($Å^3$), R0 (Å), Dro (e $Å^{-3}$)	68043, 1.615, 0.00334
Dadimodo (https://dadimodo.synchrotron-soleil.fr/)	
Starting structures	From AlphaFold2
Rigid bodies	body1 = A: 81-141<br>body2 = A: 156-400, B: 17-96
No. of generated structures	9
χ^2 range from PepsiSAXS	2.50 - 2.94

[a] (https://www.synchrotron-soleil.fr/en/beamlines/swing#paragraphes_menu_left-block-7)

Supplementary References

1. Meisburger, S. P., Xu, D. & Ando, N. *REGALS*: a general method to deconvolve X-ray scattering data from evolving mixtures. *IUCrJ* **8**, 225-237 (2021).
2. Hopkins, J. B. *BioXTAS RAW 2*: new developments for a free open-source program for small-angle scattering data reduction and analysis. *J Appl Crystallogr* **57**, 194-208 (2024).
3. Rudenko, O., Thureau, A. & Perez, J. Evolutionary refinement of the 3D structure of multi-domain protein complexes from Small Angle X-ray Scattering data. *Proceedings of the 2019 Genetic and Evolutionary Computation Conference Companion (Geccco'19 Companion)* 401-402 (2019).
4. Ma, E. et al. Rad52-Rad51 association is essential to protect Rad51 filaments against Srs2, but facultative for filament formation. *Elife* **7**, (2018).
5. Wilkins, M. R. et al. Protein identification and analysis tools in the ExPASy server. *Methods Mol Biol* **112**, 531-552 (1999).
6. Manalastas-Cantos, K. et al. <i>ATSAS 3.0</i>: expanded functionality and new tools for small-angle scattering data analysis. *J Appl Crystallogr* **54**, 343-355 (2021).
7. Jumper, J. et al. Highly accurate protein structure prediction with AlphaFold. *Nature* **596**, 583-589 (2021).
