# Supplementary material for: A large C-terminal Rad52 segment acts as a chaperone to Form and Stabilize Rad51 Filaments
Source: Nat Commun. 2025 Jul 1;16:5589. doi: 10.1038/s41467-025-60664-x (PMC12218292; doi:10.1038/s41467-025-60664-x)
Supplement: Supplementary file 2 — Description of Additional Supplementary Files [file 41467_2025_60664_MOESM2_ESM.pdf]

Description of Additional Supplementary Files

File Name: Supplementary Data 1

Description: *S. cerevisiae* strains.
