# Supplementary material for: A large C-terminal Rad52 segment acts as a chaperone to Form and Stabilize Rad51 Filaments
Source: Nat Commun. 2025 Jul 1;16:5589. doi: 10.1038/s41467-025-60664-x (PMC12218292; doi:10.1038/s41467-025-60664-x)
Supplement: Supplementary file 4 — Reporting Summary [file 41467_2025_60664_MOESM4_ESM.pdf]

Corresponding author(s): Eric Coïc
Françoise Ochsenbein
Angela Taddei

Last updated by author(s): Apr 5, 2025

Reporting Summary

Nature Portfolio wishes to improve the reproducibility of the work that we publish. This form provides structure for consistency and transparency in reporting. For further information on Nature Portfolio policies, see our Editorial Policies and the Editorial Policy Checklist.

Statistics

For all statistical analyses, confirm that the following items are present in the figure legend, table legend, main text, or Methods section.

n/a	Confirmed	
☐	☒	The exact sample size (n) for each experimental group/condition, given as a discrete number and unit of measurement
☐	☒	A statement on whether measurements were taken from distinct samples or whether the same sample was measured repeatedly
☐	☒	The statistical test(s) used AND whether they are one- or two-sided<br>*Only common tests should be described solely by name; describe more complex techniques in the Methods section.*
☒	☐	A description of all covariates tested
☒	☐	A description of any assumptions or corrections, such as tests of normality and adjustment for multiple comparisons
☐	☒	A full description of the statistical parameters including central tendency (e.g. means) or other basic estimates (e.g. regression coefficient) AND variation (e.g. standard deviation) or associated estimates of uncertainty (e.g. confidence intervals)
☐	☒	For null hypothesis testing, the test statistic (e.g. F, t, r) with confidence intervals, effect sizes, degrees of freedom and P value noted<br>*Give P values as exact values whenever suitable.*
☒	☐	For Bayesian analysis, information on the choice of priors and Markov chain Monte Carlo settings
☒	☐	For hierarchical and complex designs, identification of the appropriate level for tests and full reporting of outcomes
☒	☐	Estimates of effect sizes (e.g. Cohen's d, Pearson's r), indicating how they were calculated

Our web collection on statistics for biologists contains articles on many of the points above.

Software and code

Policy information about availability of computer code

Data collection	- Software Topspin 4.1 (Bruker)<br>- SWING beamline acquisition software (Thureau A, Roblin P, Perez J, J Appl Crystallogr. 2021;54:1698-710)<br>- MetaMorph (64-bit,7.10.5.476) has been used to acquire fluorescent images.

Data analysis

- Software Foxtrot 3.5.10 (provided by SWING, at Soleil https://wwwpreprod.synchrotron-soleil.fr/lignes-de-lumiere/swing)
- Software RAW 2.2.1 (version downloaded from https://bioxtas-raw.readthedocs.io/en/v2.2.1/install.html)
- Server Dadimodo https://dadimodo.synchrotron-soleil.fr
- Software Sparky 3.114 (from https://www.cgl.ucsf.edu/home/sparky/ T.D. Goddard and D.G. Kneller, UCSF downloaded from),
- Software MMseqs2 (version edb8223d1ea07385ffe63d4f103af0eb12b2058e, from https://github.com/soedinglab/MMseqs2)
- Software hhfilter (version 3.3.0, from https://github.com/soedinglab/hh-suite)
- Software MAFFT (version 7.475, from https://mafft.cbrc.jp/alignment/software/)
- Software Singularity (version V3.8.3 from https://github.com/apptainer/singularity/releases/tag/v3.8.3)
- Software ColabFold (version 1.5.2, from https://github.com/sokrypton/ColabFold, commit 3e99c44eec189ec27f6d120af851adb7ff6aa2a2)
- Software AlphaFold2-Multimer (version 2.3.1)
- Software ChimeraX (version 1.7.1 (2024-01-23) from https://www.rbvi.ucsf.edu/chimerax/download.html)

SVI Huygens has been used to deconvolve fluorescent images
ImageJ 1.53C ,open source, for macOS was used for image segmentation, skeleton extraction and analyses

For manuscripts utilizing custom algorithms or software that are central to the research but not yet described in published literature, software must be made available to editors and reviewers. We strongly encourage code deposition in a community repository (e.g. GitHub). See the Nature Portfolio guidelines for submitting code & software for further information.

Data

Policy information about availability of data

All manuscripts must include a data availability statement. This statement should provide the following information, where applicable:

- Accession codes, unique identifiers, or web links for publicly available datasets
- A description of any restrictions on data availability
- For clinical datasets or third party data, please ensure that the statement adheres to our policy

Chemical shifts of Rad52 C-terminal segment -206-471 is available on the BMRB under the ID 53001
The structural model of the complex between the globular domain of S. cerevisiae Rad51 (77-400) and the disordered C-terminus of Rad52 (310-394) is available in ModelArchive at https://www.modelarchive.org/doi/10.5452/ma-7m6mt".
Microscopy data and the source data file are available in Zenodo: 10.5281/zenodo.15149086

Research involving human participants, their data, or biological material

Policy information about studies with human participants or human data. See also policy information about sex, gender (identity/presentation), and sexual orientation and race, ethnicity and racism.

Reporting on sex and gender

Use the terms sex (biological attribute) and gender (shaped by social and cultural circumstances) carefully in order to avoid confusing both terms. Indicate if findings apply to only one sex or gender; describe whether sex and gender were considered in study design; whether sex and/or gender was determined based on self-reporting or assigned and methods used.
Provide in the source data disaggregated sex and gender data, where this information has been collected, and if consent has been obtained for sharing of individual-level data; provide overall numbers in this Reporting Summary. Please state if this information has not been collected.
Report sex- and gender-based analyses where performed, justify reasons for lack of sex- and gender-based analysis.

Reporting on race, ethnicity, or other socially relevant groupings

Please specify the socially constructed or socially relevant categorization variable(s) used in your manuscript and explain why they were used. Please note that such variables should not be used as proxies for other socially constructed/relevant variables (for example, race or ethnicity should not be used as a proxy for socioeconomic status).
Provide clear definitions of the relevant terms used, how they were provided (by the participants/respondents, the researchers, or third parties), and the method(s) used to classify people into the different categories (e.g. self-report, census or administrative data, social media data, etc.)
Please provide details about how you controlled for confounding variables in your analyses.

Population characteristics

Describe the covariate-relevant population characteristics of the human research participants (e.g. age, genotypic information, past and current diagnosis and treatment categories). If you filled out the behavioural & social sciences study design questions and have nothing to add here, write "See above."

Recruitment

Describe how participants were recruited. Outline any potential self-selection bias or other biases that may be present and how these are likely to impact results.

Ethics oversight

Identify the organization(s) that approved the study protocol.

Note that full information on the approval of the study protocol must also be provided in the manuscript.

Field-specific reporting

Please select the one below that is the best fit for your research. If you are not sure, read the appropriate sections before making your selection.

☒ Life sciences ☐ Behavioural & social sciences ☐ Ecological, evolutionary & environmental sciences

For a reference copy of the document with all sections, see nature.com/documents/nr-reporting-summary-flat.pdf

Life sciences study design

All studies must disclose on these points even when the disclosure is negative.

Sample size	For imaging experiments, sample size was empirically determined based on standard deviation between replicates.
Data exclusions	Clearly false negative colonies with 0 β-galactosidase activity were excluded from Y2H experiments. For survival experiments, contaminated plates were excluded.
Replication	Survival and co-IP experiments were repeated 2 to 3 times. Y2H data are issues from 2 to 3 independent transformations. β-galactosidase activity was measured in 4 independent colonies for each transformation.<br>Dead or out-focus cells, and contaminated cultures are excluded in quantification on living cells. This criteria was pre-established.
Randomization	Not relevant
Blinding	Not relevant

Reporting for specific materials, systems and methods

We require information from authors about some types of materials, experimental systems and methods used in many studies. Here, indicate whether each material, system or method listed is relevant to your study. If you are not sure if a list item applies to your research, read the appropriate section before selecting a response.

Materials & experimental systems

n/a	Involved in the study
☐	☒ Antibodies
☒	☐ Eukaryotic cell lines
☒	☐ Palaeontology and archaeology
☒	☐ Animals and other organisms
☒	☐ Clinical data
☒	☐ Dual use research of concern
☒	☐ Plants

Methods

n/a	Involved in the study
☒	☐ ChIP-seq
☒	☐ Flow cytometry
☒	☐ MRI-based neuroimaging

Antibodies

Antibodies used	The anti-Rad51 polyclonal antibody was produced in rabbit by Eurogentec (ordered from the EC lab). anti-FLAG monoclonal antibody, Sigma, ref F3165, lot SLBL1237V. Monoclonal goat anti-mouse Alexa800, Lifetech, Ref R05061-250, Lot 160321-50. Goat anti-rabbit IR700, Advansta, Ref 05054-250, Lot 15070256. Goat anti-rabbit IR800, Advansta, Ref 05060-250, Lot 150326-48. The anti-RPA was a gift from Vinvent Geli.
Validation	anti-Flag, Alexa800, IR700 and IR800 antibodies were validated by the manufacturer. Anti-Rad51 was validated first by western blot from WT and RAD51-deleted cell extracts. anti-Rad51, anti-RPA and anti-FLAG was also validated on Rad51, RPA and Rad52-FLAG purified proteins. Anti-RPA and anti-Rad51 also show a ChIP signals at a DSB site only after DSB-induction.

Plants

Seed stocks	*Report on the source of all seed stocks or other plant material used. If applicable, state the seed stock centre and catalogue number. If plant specimens were collected from the field, describe the collection location, date and sampling procedures.*
Novel plant genotypes	*Describe the methods by which all novel plant genotypes were produced. This includes those generated by transgenic approaches, gene editing, chemical/radiation-based mutagenesis and hybridization. For transgenic lines, describe the transformation method, the number of independent lines analyzed and the generation upon which experiments were performed. For gene-edited lines, describe the editor used, the endogenous sequence targeted for editing, the targeting guide RNA sequence (if applicable) and how the editor was applied.*
Authentication	*Describe any authentication procedures for each seed stock used or novel genotype generated. Describe any experiments used to assess the effect of a mutation and, where applicable, how potential secondary effects (e.g. second site T-DNA insertions, mosiacism, off-target gene editing) were examined.*
